# 

**Published:** 1916-07-29

**Authors:** 


### CONTENTS.

| | PAGE |
|---|---|
| **Cheap Prescribing** ... ... ... ... ... ... | 399 |
| **The Praise of Fools** ... ... ... ... ... | 400 |
| **Hospital and Institutional News** ... ... ... | 401 |
| The Mesopotamia Inquiry—Women Doctors for the Regular Army—Recognition for the Wounded—Bill for War Charities—The Hotel as a Hospital—Electric Fans for Hospitals—The Effect of the War upon Ringworm—A Second Raid on the Schools—Soldiers' Embroidery—The Limits of Dispensary Usefulness—Afforestation Work for Discharged Sanatorium Patients—Infantile Paralysis at Aberdeen—A Proposed Semi-Convalescent Home—The Threepenny Doctor Convicted—The Reform of the Funeral—The Mothers' Union—Hospitals and the August Holidays—An Eventful Year—A Battle Bed at Grimsby—A New Maternity Rest Home—The Rod as a Source of Income—The Birmingham Royal Institution for the Blind—Institutional Porters and War Service—The M.A.B. Food Bill—A Remarkable Letter—Duty-Free Spirit for Hospitals—The Seamen's Hospital Society—This Week's Drug Market. | |
| **The Alimentary Rest Treatment of Diabetes** | 407 |
| Some Details of a Promising Regimen. | |
| **A Medical Causerie** ... ... ... ... ... ... | 408 |
| **Sir Victor Horsley** ... ... ... ... ... ... | 409 |
| A Man of Genius and Personality. | |
| **The Hospital Drug Supply**... ... ... ... | 410 |
| A Word of Caution to Buyers. | |
| **The Institutional Treatment of Consumption** | 411 |
| VII.—Notes on Administration in a Sanatorium. | |
| **Parliament and the Profession** ... ... ... | 412 |
| **The Matrons' and Sisters' Department** ... | 413 |
| Names in Nursing: A Call for Definitions in Terms. | |
| Round the Hospitals. | |
| The Registration Bill—Names Must Be Given—Wounded Officers at the London—Massage Training Increased—The Silver Badge. | |
| **The Editor's Letter-Box** ... ... ... ... ... | 415 |
| The Profession and the National Health Commissioners: Incapable of Work—The Control of Lay Women in Hospitals—Children's Country Holidays Fund: Letter from the Bishop of London—Nurses' Looks. | |
| **Hospital Results in 1915** ... ... ... ... ... | 416 |
| **The Department of Modern Nursing** ... ... | 417 |
| The Radcliffe Infirmary, Oxford. | |
| **Leicester Royal Infirmary**... ... ... ... ... | 418 |
| A Satisfactory Financial Position. | |
| **What's What Column** ... ... ... ... ... | iv |
| **Matrons' and Sisters' Appointments** ... ... | iv |
| **The Institutional Worker** ... (Suppl.) | 1 |
| Home-Made Surgical Appliances.. | |

